# Nurses’ experiences of competence development in home care

**DOI:** 10.1017/S1463423626101029

**Published:** 2026-03-23

**Authors:** Liisa Ranta, Marja Kaunonen

**Affiliations:** Tampere University, Tampere, Finland

**Keywords:** competence development, home care, nurse, primary care

## Abstract

**Aim:**

The aim of this study was to describe nurses’ experiences of competence development in home care.

**Background:**

Home care services are increasingly used to support clients’ coping at home. As the number of clients with multiple diseases is growing, continuous competence development is needed.

**Methods:**

Qualitative cross-sectional study. Four registered nurses (RNs) and seven licenced practical nurses (LPNs) from one well-being services county in Finland participated in interviews. The data were analysed with thematic analysis.

**Findings:**

Three themes were found in the analysis: having adequate competence to work as a nurse in home care, being a competent and developing licenced practical nurse, and being an improving and developing registered nurse. Competence development requires continuous training. Nurses need various practises to update their knowledge and skills. Managers have an important role in supporting, organizing, and timing competence development opportunities.

## Background

### Home care

Globally, the population is aging, and the number of older people is also increasing in Europe. In 2019, 22.1 per cent of the 90.4 million older people (aged over 65 years or older) in the EU-27 were living in predominantly rural regions. (Eurostat, 2020.)

In Finland, well-being service counties are responsible for organizing healthcare, social, and rescue services, which include home care services as primary care services (Ministry of Social Affairs and Health, 2024a). Home care services include home services and home nursing (Ministry of Social Affairs and Health, 2024b). Home care services provide individual support for clients in everyday life through treatment and care, activities that promote and maintain functional capacity, and interactions at clients’ homes (Social Welfare Act, 1301/2014), and home nursing constitutes a temporary multi-professional health and medical care service (Health Care Act, 1326/2010). In 2023, 185,000 clients used home care services in Finland (Finnish Institute for Health and Welfare, 2025).

Nurses are the largest professional group in home care. A total of 71 per cent of nursing professionals were practical nurses in 2023, and the share of registered nurses (RNs) and public nurses in home care was 11 per cent (Saske *et al.*, 2024). RNs have a bachelor’s degree from a university of applied sciences, which corresponds to level-six education under the European Qualifications Framework (EQF) (EU, 2018), and licenced practical nurses (LPNs) complete vocational training deemed level-four education under the EQF (Finnish National Agency for Education, 2024). The number of professionals and clients varies by well-being services counties (Josefsson and Kehusmaa, 2022).

### Competence development

Nurses’ work in home care requires diverse competence (Rusli *et al.*, 2023; Ranta and Kaunonen, 2024; Saari *et al.*, 2025) due to changing situations and clients’ complex needs (Andersson *et al.*, 2017). To work effectively and safely, nurses need extensive clinical skills, responsibility, and work in different roles. Professional development necessitates continuous education. (Pavloff and Labrecque, 2021.) According to Gannan *et al.* (2019), professional development support is one factor in the optimization of home care nursing. It is important that nurses are active and continue professional development to ensure competent care and the quality of nursing (Mlambo *et al.*, 2021).

Work experience and education level, working environment, critical thinking, adherence to professionalism, and personal factors have been identified as the factors which influence nurses’ competence development (Rizany *et al.*, 2017). However, a lack of time and a low number of staff are barriers to realizing this (Pavloff and Labrecque, 2021) in daily work.

Professional development requires management support (Gannan *et al.*, 2019), and continuing professional development should be attainable, realistic, and relevant (Mlambo *et al.*, 2021). Managers and administration play a role in organizing opportunities for nurses to enhance their skills. Therefore, they must recognize competence needs at the individual and team levels, encouraging competence development. (Andersson *et al.*, 2017.) Nurses wish that their managers support their efforts to maintain competence through continuing professional development (Rahmah *et al.*, 2022). Also, managers need opportunities for leadership development (Gannan *et al.*, 2019), and how to enable continuous professional development in a supportive environment (Mlambo *et al.*, 2021).

Nurses in home care need opportunities to develop their competencies because a lack of competence has been found to increase nurses’ stress and insecurity (Silverglow *et al.*, 2022). Competence development requires both training (Andersson *et al.*, 2017; Aune *et al.*, 2019) as well as the ability to develop (Andersson *et al.*, 2017; Claesson *et al.*, 2020), which includes willingness and opportunities to share competencies (Andersson *et al.*, 2017). Training must be standardized, constantly available, and regular, occurring at various career stages and ranging from job orientation to ongoing training (Ganna *et al.*, 2019). Competence development occurs in discussions with other health care professionals (Rusli *et al.*, 2022) and clients and their relatives (Ranta and Kaunonen, 2024), but also by means of preceptorships and mentoring (Rizany *et al.*, 2017).

In Finland, directives of the European Union guiding education of RN (EU, 2005) and the Vocational Qualification in Social and Health Care is completed by LPN (Finnish National Agency for Education, 2025). According to Chayati and Adellia (2023), the inclusion of sufficient theory and clinical practice in home care nursing in education supports interest in and understanding of home care as a working environment. Kiljunen *et al.* (2019) have defined nurses’ competence in the context of care and nursing homes in Finland, but only one study concerning the home care context was found, which reported nurses’ self-reported competencies (Grönroos and Perälä, 2008). It is important to collect information about nurses’ experiences of competence development because their competence enhances the quality of nursing care (Rahmah *et al.*, 2022) in home care. Both professional groups, RNs and LPNs, were included because they tend to work together as a team, and more information was needed about these professionals’ experiences individually. Moreover, their job descriptions are not similar. LPNs work every day of week around the clock closely with clients and RNs work include responsible for all clients’ care and they work principally weekdays.

The aim of this study was to describe nurses’ experiences of competence development in home care.

The research questions were:

1. What competencies do nurses have in relation to competence requirements in their work in home care?
2. What kind of competence development is needed in home care nursing?

## Methods

### Design

The qualitative design was selected for this study. Semi-structured individual interviews were used for data collection to explore participants’ experiences of phenomena (Sandelowski, 2000). The interview method was selected because it is a flexible (Kallio *et al.*, 2016) and effective technique for exploring the interviewees’ experiences and perceptions (Naz *et al.*, 2022).

The thematic analysis method was applied to analyse the data. The Consolidated Criteria for Reporting Qualitative Research (COREQ) (Tong *et al.*, 2007) were used for reporting the results.

### Context, sampling, and data collection

The context of this study was home care units in one well-being services county in Finland. In 2022, the well-being services county employed 239 nurses in home care (Josefsson and Kehusmaa, 2022). The county offers services in both urban and rural regions, and home care services include both telehomecare and home visits.

Previous knowledge was used to formulate questions for the interview tool. An adequate understanding of the subject was formed (Kallio *et al.*, 2016) based on literature reviews about competence needs and research on competence development. This knowledge was the basis for the creation of the interview guide. The two main themes in the interviews were the nurses’ experiences of current competence in relation to the demands of the job and competence development (Table 1). Follow-up questions were used to investigate the opportunities for competence development, competence development practices, and competence needs anticipating changes in work. The authors discussed and agreed on the interview questions. The first two interviews served as a pilot, and the obtained results indicated that there was no need to revise the interview guide. These interviews were included in the data.

**Table 1.** Interview guide

Theme	Questions
Current competence	How would you describe your competence in relation to the job requirements? What relevant competence do you believe you have for your work? What kind of competence do you need more of? What would you like to develop?
Competence development	Could you tell me how you maintain and develop your competence? How can the competence required in your work be secured in your work community? What about at the level of individual employees? What kinds of competence development practices are there in your work community? How are changes in work anticipated in the competence needs?

According to the inclusion criteria, the nurses had worked in home care services for a minimum of two months, and they held a degree as a RN or a LPN. The interviewees were recruited from all home care units of the studied county through a contact person. Information about the research was sent to the contact person, who forwarded it to nurses working in home care by email. Those nurses who were willing to participate in the study were invited to contact the first author by email. The author sent information about the research to the nurses. Moreover, a second email was sent, reminding the recipients of the opportunity to participate in the study.

Data were collected in autumn 2022. The interviews were conducted in Finnish and recorded using a voice recorder. Only the interviewee and the researcher (LR) were present in the interview situation. Neither of the researchers knew the study participants beforehand. Both researchers have experience working in primary care, but not in home care. However, they have conducted research in the field of home care.

### Data analysis

Thematic analysis followed Braun and Clarke’s six phases: familiarizing yourself with your data, generating initial codes, searching for themes, reviewing themes, defining and naming themes, and producing the report (Braun and Clarke, 2006). In the analysis process, a codebook (Braun and Clarke, 2021) and notes (Vaismoradi *et al.*, 2016) were used to support the analysis.

Familiarization with the data began during the interviews and continued during the transcription phase (Braun and Clarke, 2006). The interviews were transcribed verbatim. The transcripts included words and sounds, excluding nonverbal communication. The data were considered inductively according to the themes of the interview and subsequently coded (Braun and Clarke, 2006). Both latent and manifest content were used in the data analysis. Each quotation from the interview was assigned a number.

The different codes were sorted into potential themes, which were formed by thematic maps (Braun and Clark, 2006). The thematic maps were visualized manually based on themes, codes, and their relationships using PowerPoint and Whiteboard software.

The identified themes were named and reported (Braun and Clarke, 2006) in connection with the nurses’ competence experiences and developing needs. The authors discussed interpretations and the construction of themes until consensus was reached.

## Findings

The interviews were held over the phone (*n* = 4) and with online meeting software (*n* = 7), considering the interviewees’ expectations. The interviews were lasted between 31 and 48 minutes. A total of 11 nurses, four RNs and seven LPNs participated in the interviews. The interviewees were women aged between 33 and 61 (*n* = 10, one unknown). They had between 3 and 38 years (*n* = 11) of work experience in health care and between one year and 38 years in home care.

Three themes were generated based on the analysed data: (1) Having adequate competence to work as a nurse in home care, (2) Being a competent and developing licenced practical nurse, and (3) Being an improving and developing RN (Table 2).

**Table 2.** Main themes and subthemes

Main themes	Having adequate competence to work as a nurse in home care	Being a competent and developing licenced practical nurse	Being an improving and developing registered nurse
**Subthemes**	Identifying their own competence	Developing their competence at different stages of their career	Competence development is a part of daily work
	Identifying necessary skills	Development opportunities and practises	Opportunities and practises to develop one’s competence
	Competence needs related to changes in work and supplementing related competence	Competence management in their work community	A role in supporting learning and developing competence in their own team
		Developing competence management at the organizational level	

### Having adequate competence to work as a nurse in home care

The nurses felt that adequate competence consists of *identifying their own competence*, *identifying necessary skills, competence needs related to changes in work, and supplementing related competence.*

Both RNs and LPNs described that they had adequate competence to work in home care. They had the skills to work alone. They had to trust their own expertise and value their own competence. The LPNs mentioned that their competence had not been adequate early on in their careers. When nurses start their work as new graduates, they do not have sufficient skills to work alone in home care.

‘There was a feeling that I can’t do anything. I am a graduated LPN, but when you go to work, you really don’t know what kinds of clients you will meet, what kinds of needs they will have, what you will have to do there (in their homes)’. (Participant 7)

It was important to recognize the need for continuously updating knowledge as work changes. Nurses had to have a desire to develop their own competence, and they had to be able to do it.

‘It is essential that you are able to ask someone or look for the information somewhere else’. (Participant 11)

The LPNs described that changes in home care may lead to a decline in knowledge. As an example of this, they described medical robots used to administer single-dose medicine pouches for patients, which reduced the nurses’ medical knowledge. They also needed to update their licences related to various topics regularly, e.g. their medicine licences. For this purpose, the LPNs updated their knowledge independently. They also mentioned the need for updating knowledge related to information retrieval skills, technological skills, and nursing practice skills. Participants in this study reported using the Google search engine when having a need for quickly available information in the field. Additionally, they used evidence-based internet databases, such as a portal for healthcare professionals, when they had more time, for example, during office time. New treatments may be suddenly introduced to clients, and there is not enough time to gather in-depth information. Calling a colleague and asking them for help is the most commonly used information source.

The RNs felt that it was important to know the limitations of one’s own competence, value one’s competence, and have the skills to support other nurses in a team. Continuous learning required a positive attitude and a development-oriented mindset, but also the willingness to perform work in a new way and have an interest in learning in changing situations. The nurses felt they needed more skills in leadership and coaching a team, technological skills, and knowledge of their nursing speciality.

‘I maintain my competence by studying leadership and development (degree)… and we have a lot of training (in the organization), which gives us the opportunity to deepen our knowledge and learn new things… but I feel that you must take responsibility for developing your own competence…you have the responsibility like in self-leadership’. (Participant 4)

### Being a competent and developing licenced practical nurse

The LPNs described that being a competent and developing nurse included *developing their competence at different stages of their career, development opportunities and practices, competence management in the work community, and developing competence management at the organizational level.*

They mentioned that some lack sufficient knowledge and skills at the beginning of their career, while some nurses do not want to develop their competence at the later stages of their careers. While it was considered important to have adequate competence for fulfilling daily work requirements, they had also noticed a need for continuous development. There is a need to recognize the lack of knowledge and motivation to develop one’s skills.

The opportunities and practices to develop one’s competence the LPNs mentioned included training while working, learning with support in a multi-professional team, information retrieval, and learning alone. This required taking responsibility and having the motivation for developing one’s competence and arranging a situation where learning is possible.

‘If someone has completed some sort of training, they can talk about it in weekly team meetings’. (Participant 1)

Developing competence management in the work community necessitates information and communication about training possibilities at the same place, monitoring nurses’ competence and development needs, but also anticipating these needs. The nurses felt that these were good practises and that nurses have responsibilities in the working community and participate in training regularly. They also had expectations of competence management at the organizational level. They mentioned that they wished for enough time and support to develop their competence. All nurses must be given similar opportunities to develop their competence. Continuous changes at work necessitate recognizing new competence requirements as well as an open and participatory approach and joint discussions.

‘There is some training we have to participate but we don’t have many resources. Another opportunity is to participate in training on your own time. I try to study by myself a bit, find information on the internet, and read training materials, for example, on medicine-dispending robots. We have short trainings, handouts and inductions, but you can also ask colleagues’. (Participant 8)

### Being an improving and developing registered nurse

The RNs felt that *competence development is a part of daily work*. Competence development was realized through work and discussions. Professional competence advances as nurses make progress in their careers. The RNs’ role involved recognizing a lack of competence in their team and supporting and advising other team members.

‘Of course, there’s the registered nurse’s degree, but experience in clinical competence plays a big role in professional competence, as does, to some extent, intuition. For intuition, you need experience in working in the field and combining knowledge with obtained information, and to read evidence-based information and combine it to practice’. (Participant 2)

Regarding the *opportunities and practices to develop one’s competence*, RNs mentioned the same practices as LPNs; training while working and learning in a multi-professional team. They needed skills in information retrieval and independent learning. They emphasized the importance of taking responsibility to develop one’s knowledge and skills at work.

They also felt that they had *a role in supporting learning and developing competence in their own team*. This included enabling the maintenance of professional skills in the team, supervising the fulfilment of regularly required training, maintaining and enabling information sharing in the team, and motivating nurses to competence development.

‘Self-management could be something in this job that should perhaps be used instead of some training… now, the focus is often on professional know-how, but with a lot of pressure. Maybe that could be required more rather than just knowledge on diabetes’. (Participant 9)

‘Of course, I recognize people who are not interested in developing their own competence. Then we ask the team to motivate them. I feel that sense of community is important when we know that someone has a challenge or is not interested’. (Participant 4)

## Discussion

### Main findings

This study described that competence development requires input from managers, home care teams, and nurses themselves. Managers need to know and understand which competence requirements are necessary in home care and which competencies individual nurses and teams must still develop (Andersson *et al.*, 2017). Competence development requires knowledge management from managers to determine and anticipate competencies (Karskikas *et al.*, 2025). There is also a need for organizational core capabilities and future knowledge (Probst, 1998), assessment of competencies, possible competence development, and management of competence culture (Karsikas *et al.*, 2025). Changing situations in daily work and clients with multiple health problems require nurses to continuously update their knowledge and skills (Rusli *et al.*, 2023; Ranta and Kaunonen, 2024). In this study, the nurses felt that they had adequate knowledge and skills to work in home care, but that continuous development was needed; for example, the deployment of new technological solutions required more knowledge in technology. Also, the need for professional and evidence-based knowledge required information retrieval skills. Pavlov and Labrecque (2021) reported that one of the barriers to continuous learning was a lack of skills in conducting literature searches.

Developing competence requires knowledge identification to support employees in their knowledge-seeking activities (Probst, 1998) and managers’ competence in system management, including tools, processes, and information systems (Karsikas *et al.*, 2022). The evaluation of competence needs can be supported by knowledge maps (Probst, 1998) or assessment tools. While there was development of competence assessment tools for home care nurses, according to Rusli *et al.* (2024) there is a need for an instrument encompassing all the relevant competencies.

Some nurses in this study saw their managers at performance appraisals where they could discuss their own developing needs. The home care nurses felt that it should be noted that they had different responsibilities for their clients and their care compared to those working in a hospital ward, because they work alone. It is important to note that the move of a nurse to caring for clients at their homes and supporting their life at home for as long as possible needs to be supported by education (Pavlov and Labrecque, 2021). The nurses in our study felt that learning with the support of a multi-professional team was one of the practises that allowed them to develop competence. They have an opportunity to consult different professionals in client care, e.g. pharmacists, physiotherapists, and medical doctors. These opportunities are important because meetings with other professionals are known to increase competence development (Rusli *et al.*, 2023).

Lacking the time to update one’s knowledge is common. The nurses interviewed for this study reported that while time was reserved for training by their shift planner, this often ended up being cancelled. One nurse explained that a lack of staff and a growing number of clients are prone to lead to cancelling plans to participate in training and missed learning opportunities. Sometimes home nurses study new nursing practices on the way to a client’s home, as the daily work includes a lot of home visits, and there is no extra time. Other studies have also mentioned that the lack of time hinders competence development and acknowledged the need for workplace scheduling (Pavloff and Labrecque, 2021). Work carried out in urban and rural areas requires different time planning. Distances between clients’ homes and weather conditions are also reasons for changing situations.

Therefore, it is important to find different solutions to allow nurses to develop their competence alongside traditional forms of training. Education and training enabling competence development need to be provided in various formats using face-to-face, online learning, simulation, or traditional learning (Pavloff and Labrecque, 2021). In this study, the nurses described that development must occur as a part of daily work. The team’s role in supporting competence development was recognized as important. The nurses felt that RNs have a role in supporting the nurses in their team to develop their competence and update their knowledge. While leadership supports collaboration and enables capacity building (Gannan *et al.*, 2019), according to Rizany *et al.* (2017), nurse managers also need to support nurses in education.

At the time of the interviews, there were ongoing organizational changes in the healthcare and social welfare services in Finland. The newly established well-being service counties combine services previously provided by municipalities, cities, and municipal federations. The nurses felt that some home care units had more information and knowledge of the changes and new service solutions than others. They expected everyone to have identical opportunities for developing their competence and obtaining information.

The nurses described that they had lacked adequate competence to work in home care early on in their careers. Furthermore, it was reported earlier that senior nurses’ long work experience and more learning opportunities might result in better competencies (Rizany *et al.*, 2017). Clients in home care typically have multiple diseases and treating their health conditions is becoming increasingly demanding (Sanerma *et al.*, 2022). Saari *et al.* (2025) determined a total of 79 competency statements in the context of advanced home care in Canada. Some new competencies were detected: trauma-informed care, data-driven decision-making, and provision of culturally safe care, which are relevant elements to integrate into the home care environment in education and training.

Work in home care was described as attractive, because it offers autonomy, freedom, and work in a self-directed team. Patient situations varied and nurses served as ‘linchpins’ in their communities and client care. It is also important to take this description into consideration in education and practice (De Groot *et al.*, 2018) to support nurses and nurse students to gather and develop these competencies.

### Limitations

The concept of competence development has not been clearly defined. The concepts of knowledge management, competence management, and different concepts have been used to understand competence development and management in the contexts of leaders and managers, as indicated in previous studies (Probst, 1998; Karsikas *et al.*, 2002; Myllymäki *et al.*, 2022). The present study described the experiences of nurses in competence development and their expectations for their managers and employers.

This study included nurses’ experiences gathered only from one well-being services county in Finland, and in the interviews, it was recognized that the practises of competence development differed between regions and organizations in the county. As a result, the interviews provided information only from these regions where the participants were working. In utilizing the results, it is important to acknowledge that there are differences in organizing home care services not only between but also within countries. As a result, one must be careful when applying these results to other home care units, especially in other countries.

As only four RNs participated in the interviews, it is important to gather more information about the experiences of RNs. Even though the competence requirements set for RNs and LPNs were rather similar, it is important to explore RNs’ experiences more thoroughly.

## Conclusion

Nurses working alone in home care need extensive competence and continuous development. Home care units have various roles to enable this. It is important to define these roles in each unit.

Opportunities to update knowledge and skills in different ways enable competence development. In this context, managers have an important role in organizing, reporting, and timing them. A shift planner includes training opportunities in home nurses’ daily work schedules.

In the nurses’ work teams, it is important to recognize the competence requirements of individual team members and enable continuous competence development. The RNs serving as team leaders have the duty to pay attention to team members’ competence needs, supporting development, and ensuring participation in regular training. The work of nurses also requires adequate knowledge. They need to pay attention to their own developing needs, consult others, and gather information. They must be motivated to develop their competence.

Caring for increasingly seriously ill clients in home care requires wide knowledge and skills from the nurses. Nurses have different competence development needs in various stages of their careers. It is important to recognize this in home care units but also in nursing education and the provision of continuing education.

In the future, more research on competence development in home care is needed, as information on competence development practices, roles, and competence development is needed in different career stages.

## Supporting information

Ranta and Kaunonen supplementary material 1 Ranta and Kaunonen supplementary material

Ranta and Kaunonen supplementary material 2 Ranta and Kaunonen supplementary material

## Data Availability

Data available on request from the authors.

## Supplementary material

The supplementary material for this article can be found at https://doi.org/10.1017/S1463423626101029.

## References

[ref1] Andersson H , Lindholm M , Pettersson M and Jonasson L-L (2017) Nurses’ competencies in home healthcare: An interview study. BMC Nursing 16(65), 1–8. 29176934 10.1186/s12912-017-0264-9 PMC5693583

[ref2] Aune E and Struksnes S (2019) Home care nurses’ experience of providing health-care to patients with hard-to-heal wounds. Journal of Wound Care 28(3), 178–187. 30840548 10.12968/jowc.2019.28.3.178

[ref3] Braun V and Clarke V (2006) Using thematic analysis in psychology. Qualitative Research in Psychology 3(2), 77–101.

[ref4] Braun V and Clarke V (2021) Can I use TA? Should I use TA? Should I not use TA? Comparing reflexive thematic analysis and other pattern-based qualitative analytic approaches. Counselling and Psychotherapy Research 21, 37–47. 10.1002/capr.12360.

[ref5] Chayati N and Adellia R (2023) Understanding of home care and interest level of nursing students working as home care nurses. Bali Medical Journal 12(1), 506–509. 10.15562/bmj.v12i1.3737.

[ref6] Claesson M , Jonasson L-L , Lindberg E and Josefsson K (2020) What implies registered nurses’ leadership close to older adults in municipal home health care? A systematic review. BMC Nursing 19, 30. eCollection. 32336946 10.1186/s12912-020-00413-1 PMC7171838

[ref7] De Groot K , Maurits EEM and Francke AL (2018): Attractiveness of working in home care: An online focus group study among nurses. Health & Social Care in the Community 26(1), e94–e101. https://doi-org.libproxy.tuni.fi/10.1111/hsc.12481. 28730631 10.1111/hsc.12481

[ref8] EU (2005) Official Journal of the European Union DIRECTIVE 2005/36/EC OF THE EUROPEAN PARLIAMENT AND OF THE COUNCIL Available at https://eur-lex.europa.eu/legal-content/EN/TXT/HTML/?uri=CELEX:32005L0036 (accessed 15 October 2025).

[ref9] EU (2018) Council Recommendation of 26 November 2018 on promoting automatic mutual recognition of higher education and upper secondary education and training qualifications and the outcomes of learning periods abroad. Document 32018H1210(01). Available at https://eur-lex.europa.eu/legal-content/EN/TXT/?uri=CELEX:32018H1210(01) (accessed 15 October 2025).

[ref10] EUR-Lex - 32016R0679. Regulation (EU) 2016/679 of the European Parliament and of the Council of 27 April 2016 on the protection of natural persons with regard to the processing of personal data and on the free movement of such data, and repealing Directive 95/46/EC (General Data Protection Regulation). Available at https://eur-lex.europa.eu/legal-content/EN/TXT/?uri=CELEX%3A32016R0679 (accessed 15 October 2025).

[ref11] Eurostat (2020) Ageing Europe - statistics on population developments. Available at https://ec.europa.eu/eurostat/statistics-explained/index.php?title=Ageing_Europe_-_statistics_on_population_developments (accessed 15 October 2025).

[ref12] Finnish Institute for Health and Welfare (2025) Homecare 2025. [In Finnish: Kotihoito 2023]. Updated on 29 April 2025. https://thl.fi/tilastot-ja-data/tilastot-aiheittain/ikaantyneet/kotihoito.

[ref13] Finnish National Agency for Education (2024) Qualifications frameworks. https://www.oph.fi/en/education-and-qualifications/qualifications-frameworks (accessed 15 October 2025).

[ref14] Finnish National Agency for Education (2025) Vocational Qualification in Social and Health Care. Competence area of Care and Rehabilitation for Elderly People. https://eperusteet.opintopolku.fi/#/en/ammatillinen/3689879/tekstikappale/6660572 (accessed 15 October 2025).

[ref15] Ganann R , Weeres A , Lam A , Chung H and Valaitis R (2019) Optimization of home care nurses in Canada: A scoping review. Health & Social Care in the Community 27(5), e604–e621. 31231890 10.1111/hsc.12797 PMC6851676

[ref16] Grönroos E and Perälä M-L (2008) Self-reported competence of home nursing staff in Finland. Journal of Advanced Nursing 64(1), 27–37. 10.1111/j.1365-2648.2008.04747.x. 18808590

[ref17] Health Care Act (1326/2010) Home nursing. 25 §. [In Finnish: Kotisairaanhoito]. https://www.finlex.fi/eli?uri=http://data.finlex.fi/eli/sd/2010/1326/ajantasa/2025-06-27/fin (accessed 15 October 2025).

[ref18] Josefsson K and Kehusmaa S (2022) Home care in May 2022. [In Finnish. Kotihoito toukokuussa 2022: vain puolet kotihoidon työajasta on asiakasaikaa]. Finnish Institute for Health and Welfare. http://urn.fi/URN:ISBN:978-952-408-007-1 (accessed 15 October 2025).

[ref19] Kallio H , Pietilä AM , Johnson M and Kangasniemi M (2016) Systematic methodological review: Developing a framework for a qualitative semi-structured interview guide. Journal of Advanced Nursing 72(12), 2954–2965. 27221824 10.1111/jan.13031

[ref20] Karsikas E , Meriläinen M , Tuomikoski AM , Koivunen K , Jarva E , Mikkonen K , Oikarinen A , Kääriäinen M , Jounila-Ilola P and Kanste O (2022) Health care managers’ competence in knowledge management: A scoping review. Journal of Nursing Management 30(5), 1168–1187. 10.1111/jonm.13626. 35403311 PMC9542587

[ref21] Karsikas E , Meriläinen M , Koivunen K and Kanste O (2025) Health and social care managers’ self-assessed competence in knowledge management: A descriptive cross-sectional study. Journal of Advanced Nursing 81(2), 787–797. 10.1111/jan.16237. 38752616 PMC11729214

[ref22] Kiljunen O , Välimäki T , Partanen P and Kankkunen P (2019) Older people nursing in care homes: An examination of nursing professionals’ self-assessed competence and its predictors. International Journal of Older People Nursing 14, e12225. 10.1111/opn.12225. 30729686

[ref23] Mlambo M , Silén C and McGrath C (2021) Lifelong learning and nurses’ continuing professional development, a metasynthesis of the literature. BMC Nursing 20, 62. 33853599 10.1186/s12912-021-00579-2 PMC8045269

[ref25] Ministry of Social Affairs and Health (2024a) Primary healthcare. Ministry of Social Affairs and Health. https://stm.fi/en/primary-health-care (accessed 15 October 2025).

[ref26] Ministry of Social Affairs and Health (2024b) Services and benefits for old people. Ministry of Social Affairs and Health. https://stm.fi/en/older-people-services/services-and-benefits (accessed 15 October 2025).

[ref27] Myllymäki S , Laukka E and Kanste O (2022) Health and social care frontline leaders’ perceptions of competence management in telemedicine in Finland: An interview study. Journal of Nursing Management 30(7), 2724–2732. 10.1111/jonm.13740. 35852809 PMC10087294

[ref28] Naz N , Gulab F and Aslam N (2022) Development of qualitative semi-structured interview guide for case study research. Competitive Social Sciences Research Journal 3(2), 42–52.

[ref29] Pavloff M and Labrecque ME (2021) Continuing education for home care nurses: An integrative literature review. Home Healthcare Now 39(6), 310–319. 34738966 10.1097/NHH.0000000000001005

[ref30] Probst GJ (1998) Practical knowledge management: A model that works. PRISM-CAMBRIDGE MASSACHUSETTS- 17–30.

[ref31] Rahmah NM , Hariyati Rr TS and Sahar J (2022) Nurses’ efforts to maintain competence: A qualitative study. Journal of Public Health Research 11, 2736. 10.4081/jphr.2021.2736 PMC8941307 35244357

[ref32] Ranta L and Kaunonen M (2024) Requirements for nurses’ competencies in home care: An integrative review – a mixed-method approach. Nordic Journal of Nursing Research 44, 1–10.

[ref33] Rizany I , Hariyati Rr TS and Handayani H (2017) Factors that affect the development of nurses’ competencies: A systematic review. Enfermería Clínica 27(Suppl. Part I), 154–157.

[ref34] Rusli KDB , Ong SF , Speed S , Seah B , McKenna L , Lau Y and Liaw SY (2022) Home-based care nurses’ lived experiences and perceived competency needs: A phenomenological study. Journal of Nursing Management 30, 2992–3004. 35599299 10.1111/jonm.13694

[ref35] Rusli KDB , Tan AJQ , Ong SF , Speed S , Lau Y and Liaw SY (2023) Home-based nursing care competencies: A scoping review. Clinical Nursing 32, 1723–1737. 10.1111/jocn.16169 34897853

[ref36] Rusli KDB , Chua WL , Ang WHD , Ang SGM , Lau Y and Liaw SY (2024) A hybrid systematic narrative review of instruments measuring home-based care nurses’ competency. Journal of Advanced Nursing 80(7), 2647–2671. 10.1111/jan.15904. 37849066

[ref37] Saari M , Coumoundouros C , Tadeo J , Chyzzy B , Northwood M and Giosa J (2025) Advancing home health nursing competencies in Canada to reflect a dynamic care environment and complex population health needs: A modified eDelphi study. BMC Nursing 24(1), Article 378. 10.1186/s12912-025-03045-5. PMC11974033 40197356

[ref38] Sanerma P , Paavilainen E and Åstedt-Kurki P (2022) Differences in home-care services in Finland for older adults between 2012 and 2019 - a developmental evaluation study. Home Health Care Services Quarterly 41(4), 341–356. 10.1080/01621424.2022.2091500. 35748493

[ref39] Sandelowski M (2000) Focus on research methods. Whatever happened to qualitative description? Research in Nursing & Health 23, 334–340. 10940958 10.1002/1098-240x(200008)23:4<334::aid-nur9>3.0.co;2-g

[ref40] Saske S , Josefsson K , Karttunen T and Sorvali J (2024) State of services for the elderly 2023. [In Finnish Vanhuspalvelujen tila 2023. Kotihoidon asiakkaille suunniteltu palvelutuntien määrä toteutuu puolessa yksiköistä]. Finnish Institute for Health and Welfare. https://urn.fi/URN:NBN:fi-fe2024041317418.

[ref41] Silverglow A , Johansson L , Lidén E and Wijk H (2022) Perceptions of providing safe care for frail older people at home: a qualitative study based on focus group interviews with home care staff. Scandinavian Journal of Caring Science 36, 852–862. 10.1111/scs.13027 34423863

[ref42] Social Welfare Act (1301/2014) Home care. 19 a § (26.8.2022/790) [n Finnish: Kotihoito] https://www.finlex.fi/fi/lainsaadanto/2014/1301#chp_3__sec_19.

[ref43] The World Medical Association (2024) Declaration of Helsinki – ethical principles for medical research involving human subjects. WAM. https://www.wma.net/policies-post/wma-declaration-of-helsinki/. 19886379

[ref44] Tong A , Sainsbury P and Craig J (2007) Consolidated criteria for reporting qualitative research (COREQ): a 32-item checklist for interviews and focus groups. International Journal for Quality in Health Care 19(6), 349–357. 17872937 10.1093/intqhc/mzm042

[ref45] Vaismoradi M , Jones J , Turunen H and Snelgrove S (2016) Theme development in qualitative content analysis and thematic analysis. 10.5430/jnep.v6n5p100.

[ref46] Tampere University (2023) Responsible science and research. https://www.tuni.fi/en/research/responsible-science-and-research (accessed 19 February 2026).

